# Preoperative preparation for Graves’ disease

**DOI:** 10.3389/fendo.2023.1234056

**Published:** 2023-08-23

**Authors:** Tianfeng Xu, Xun Zheng, Tao Wei

**Affiliations:** Division of Thyroid Surgery, Department of General Surgery, West China Hospital, Sichuan University, Chengdu, China

**Keywords:** Graves’ disease, preoperative preparation, thyroidectomy, hyperthyroidism, treatment

## Abstract

Thyroidectomy is always regarded as the crucial treatment for Graves’ disease, especially in cases of poor efficacy or excessive side effects of antithyroid- drugs and 131I radioiodine therapy. To decrease the incidence of hemorrhage, thyroid storms and other severe complications during the perioperative period, surgeons explore different therapies to prepare for thyroidectomy. We performed a review of preoperative preparation with a focus on the Graves’ disease population. Most of the previous schemes are effective, which contributes to the smooth operation of patients, but there is no unified standard for preoperative preparation. This review aims to summarize the preoperative preparation of Graves’ disease and the latest developments. Prospective studies with longer follow up-up periods are required to select appropriate preoperative regimens based on personal thyroid statements and to identify target populations of benefit.

## Introduction

Graves’ disease (GD) is the most common pathogeny of hyperthyroidism, disturbing nearly 2% of women and 0.2% of men globally (1), and causing a significant threat to human physical and mental health (2). As an autoimmune disease, even though the pathogenesis is complicated and has not yet been fully clarified (3), the treatment of GD is relatively definitive, including antithyroid drugs (ATDs), radioiodine therapy (RAI), and surgery. GD patients should be well prepared preoperatively as much as possible to achieve a steady state both clinically and biochemically.

## Indications for surgery

For GD patients, whether to accept surgery is usually based on the severity of clinical symptoms of hyperthyroidism, the reactivity of other treatments, the availability of experienced surgical centers, and individual preference. The main situations recommending surgery are as follows: large thyroid nodules, diagnosed or highly suspected thyroid malignant tumor, women of reproductive age wishing for pregnant within 4 months or women in mid pregnancy (4), drug intolerance, poor efficacy, contraindicated to RAI or recurrence, large goiter with or without symptomatic compression, coexisting severe extrathyroidal diseases, etc. (5). Sometimes, surgery is considered the best treatment for GD patients because of the lowest relapse, direct healthcare costs (6) and a significantly faster biochemical recovery, for which sound preoperative preparation is an essential prerequisite (7).

## The goal of preoperative preparation

The thyroid is a blood-rich organ that is easily congested and edematous under hyperthyroidism. High serum free thyroxine (FT4) and free triiodothyronine (FT3) not only cause low or suppressed thyroid stimulating hormone (TSH), but also cause metabolic dysfunction, tachycardia, heart failure, etc, which are hidden dangers causing complications. A rare but serious complication of perioperative Graves’ disease is thyroid storm, and mortality rates between 8% and 25% are reported (8). Given the seriousness of thyroid storm and the difficulty of identifying the risk, GD patients who choose to accept surgical treatment should follow the current guidelines. In addition to thyroid storm, the possibility of bleeding, permanent hypoparathyroidism, recurrent laryngeal nerve paralysis, and other severe complications are slightly higher than that in patients who undergo thyroidectomy without hyperthyroidism (9). In brief, to sustain the stability of the internal biochemical environment and avoid life-threatening thyroid storm, careful preoperative management is urgently needed.

## Preoperative preparation for Graves’ hyperthyroidism

Over the years, surgeons have been trying to explore the optimum preoperative strategy. Classic drugs utilized in endocrine therapy for Graves’ disease play a significant part in the perioperative safety of GD. Regarding the mechanism, illustrated with examples, ATDs depress the synthesis of thyroid hormone by interfering with the iodination of thyroid peroxidase (TPO), beta-blockers mainly control the peripheral effects of excess FT4 and FT3 (5), and iodine even inhibits the generation of vascular endothelial growth factor (VEGF) (10) in serum and significantly reduces blood flow of the thyroid (11, 12). In clinical practice, the aim of making GD patients prepare preoperatively is usually achieved with different combinations of agents having the effects mentioned above.

Commonly, if the surgery is not arranged in emergency and GD patients without contraindication or severe side effects, ATDs are primarily recommended as a fundamental treatment. Lugol’s solution was created in 1829 and consisted of inorganic iodine (5%), potassium iodide (KI, 10%) and distilled water (13). Iodine solution has been utilized as an adjuvant therapy in GD patients preoperatively for almost a century; beta-blockers are used to alleviate the symptoms of hyperthyroidism, and they can benefit cardiac function. Ultimately, the choice of preoperative preparation is decided by the time available for preoperative preparation, the volume of a diffusely enlarged thyroid, the clinical manifestation of hyperthyroidism and the curative effect of previous treatments.

## The regimen of pharmacologic agents

### Lugol’s solution or other iodine solution

Originally, Lugol’s solution was used as a treatment for tuberculosis, disinfectants and test kits for starch. Until the 1920s, Lugol’s solution was formally given to Graves’ disease patients as a standard preoperative preparation (13). Through the Na+/I symporter, excess iodine is transported to the thyroid, inhibiting TPO and decreasing the synthesis of thyroid hormones (14).

Even though iodine is used singly, the dosage and usage are also different; the common duration is approximately 10 days for the escape phenomenon (14) from Wolff-Chaikoff is expected to probably appear 10 days later. Using Lugol’s solution preoperatively resulted in a 7.4x reduction in the rate of intraoperative blood loss (15) compared with the control group. A randomized controlled trial (RCT) showed that the mean blood loss was significantly different between the experience group, which was given 8 drops of saturated potassium iodide solution (SSKI) for 7 days, and the control group (p=0.036) (16). Taking 1 drop containing 0.05 g potassium chloride (KI) ter in die (tid) orally for 10 days prior to operation, the incidence of transient hypoparathyroidism (P = 0.018) and transient hoarseness (P = 0.009) were decreased with the matched group (17). GD patients who accepted potassium iodide solution of three drops bis in die (bid) for 13 days of treatment (18) had an obviously longer operation time (p<0.001) and heavier weight of the thyroid gland (p<0.001), which indicates that this group probably has a higher degree of difficulty, while the occurrence of postoperative vocal cord paresis (temporary vocal cord paralysis, p=0.082) was significantly decreased.

In the past 30 years, iodine interventions have varied from 0.3 ml tid for 9 days, 20 drops quaque die (qd) for 10-14 days, 10 drops qd for 10 days, 0.8 mg/kg qd for 10 days, to 8 drops qd for 7days,1 drop tid for 10 days and 7 drops qd for 10 days (19, 20). However, in GD patients, the application of iodine before surgery is still the subject of big debate. Iodine solution indeed decreases the vascularity of the thyroid and ultimately reduces intraoperative blood loss, but it is not equivalent to decreasing postoperative complications; in other words, its advantages sometimes cannot be reflected in clinical perioperative results. Research has confirmed that regarding the main postoperative complications, there were no significant differences between the SSKI group and the group without SSKI: the incidence of transient hypoparathyroidism was 40.9% versus 50%, while the incidence rate of permanent hypoparathyroidism was 6.8% versus 13%,which may attribute to the adhesion of surrounding tissues, that of transient recurrent laryngeal nerve (RLN) palsy was 2.3% versus 8.7%,and that of definitive RLN palsy was 2.3% versus 2.2% (21, 22). Furthermore, iodine causes adhesion to the surrounding tissue through fibrotic reactions, which makes it harder to position and protect the parathyroid gland during thyroidectomy, which increases the incidence of hypoparathyroidism (20). Other reasons for refusing to use iodine before surgery are mainly due to anxiety about side effects, including intolerance, digestive tract reaction, allergy, rash, neck indisposition or painless thyroiditis caused by iodine (23). These controversies could be understood, for negative results may be attributed to the thyroid volume of GD enrolled patients in research, or the hyperthyroidism severity of those patients is mild to moderate. For such patients, euthyroidism is not required preoperatively, and the operation can be carried out safely in hyperthyroidism.

Effective preoperative preparation still consists of iodine solution considering its advantages. Examples and effects of different applications of iodide as preoperative preparations are summarized in **Table 1**. More prospective research on GD patients with severe, under-controlled hyperthyroidism is needed to confirm the target GD patients who are suitable to accept iodine preoperatively and elaborate the necessity of iodine preparation before surgery.

**Table 1:** Examples and effect of different applied doses of iodide preoperative preparations in the treatment of Graves’ disease in different publications.

Year of publication	Literature	Participants	Iodide mg/day	Treatment duration	Iodine/ in total	Outcomes
2022 (24)	Guideline	/	unknown	7-14 days	unknown	FT3 normalized
2021 (22)	Retro	F:41/44, ¯y: 46.9	37.5mg or 60mg	10 days or 8 days	375mg or 480mg	No difference
2020 (18)	Retro	F:108/125, ¯y:37	180mg	13 days	2340mg	operation time ↓**
2018 (17)	Pro	F:22/28, ¯y: 38		10 days	1500mg	THP↓*
2017 (16)	RCT	F:14/18, ¯y:39.44		7 days	2800mg	blood loss ↓*
2017 (25)	Retro	F:25/27, ¯y:39	mean 100.5mg	7-10 days	168mg-2110mg	FT3↓* FT4↓**
2017 (10)	Pro	Total:25, ¯y: 32.2	190mg	10 days	1900mg	blood flow↓*
2016 (26)	guideline	/	120-300mg	10 days	1200-3000mg	recommended
2016 (15)	RCT	F:16/20 ¯y:38.69	0.8mg/kg	10 days	unknown	Blood loss 7.40x↓
2007 (27)	Pro	F:14/17, ¯y:32.2	190mg	10 days	1900mg	Blood loss 9.33x↓

The total dosage of iodine was calculated from the corresponding solution used in research.

F, female; ¯y, mean or median age; FT3, Free triiodothyronine; FT4, Free thyroxine; RCT, Randomized Controlled Trial; Retro, Retrospective study; Pro, Prospective research; THP, transient hypoparathyroidism;↓, decrease significantly; *:p<0.05, **:p<0.001, 7.40x:7.40-fold, 9.33x: 9.33-fold.

### ATDs, iodine solution

In addition to the previous experience of using a certain kind of drug mentioned above singly, surgeons made some exploration of combined regimens of medicine, some of which obtained satisfactory results.

ATDs are empirically considered basic medicine for the long-term control of hyperthyroidism. However, sometimes ATDs used singly are not enough for those patients with moderate to severe hyperthyroidism. Additional iodine solution treatment for ten days is beneficial to preoperative preparation by decreasing thyroid vascularity and minimizing surgical blood loss (28). When a patient develops side effects of ATDs or who relapses and is still thyrotoxic, iodine is an excellent means of rendering the toxic patient’s euthyroid for its Wolff-Chaikoff effect (29).

Even though this combination mainly concluded from practice lacks adequate statistical data, the three-step regimen was commonly acknowledged as effective, including giving ATDs to establish a euthyroid state first, and then deciding whether to use beta-blockers and the dosage according to the severity of individual hyperthyroidism. Iodine therapy is not a routine treatment for most patients, but it is also an important means for some patients with poor preoperative preparation for ATD. For these patients, once the operation time is determined, iodine can be applied 7-8 days before the operation date (30).

### ATDs and levothyroxine sodium

In addition to the regimen of ATD combined with iodine, Chinese scholars have also proposed other schemes. ATDs can control hyperthyroidism but increase TSH, which promotes the generation of goiters to a certain extent and increases the difficulty of surgery. Levothyroxine sodium is often utilized as replacement therapy for hypothyroidism, which can also decrease TSH via negative feedback of the pituitary thyroid axis. These two drugs may complement each other to maintain stable thyroid function. Based on this, Zhu Jing-qiang et al. (12) designed a study in which patients were divided into different groups on the basis of the dosage and duration of drugs prepared before operation in a double-blind design. Furthermore, the treatment group was divided into 4 subgroups: A (60 cases), B (61 cases), C (64 cases) and D (59 cases). Phase I: Patients were given propylthiouracil 450-900mg/d or methimazole 45-90mg/d. Phase II: continue to take antithyroid drugs plus levothyroxine(L-T4) 1.6 μg/kg or thyroxine tablets 20-60mg/d. For group A, the time of phase II was 2-4 months, and group B halved. The dosage of ATDs was reduced to propylthiouracil 150-300mg/d or methimazole 15-30mg/d in group C. In contrast, group D adjusted the time of phase II to 1-2 months, maintaining the same dosage of ATDs as group C. The results showed no significant difference in blood loss between group D and the control group, which was more significant than that in the other treatment groups (control group (324.76 ± 163.26) ml, group A (195.74 ± 57.07) ml, group B (230.00 ± 70.81) ml, group C (240.47 ± 80.29) ml, group D (314.75 ± 96.46) ml, P<0.05).They also drew a similar conclusion to predecessors that group A (sufficient ATDs and L-T4) is closer to the histologically positive pathological changes of normal thyroid tissue. They named this preoperative treatment the “preoperative preparation method of sequential thyroid defunctionalization”.

In another clinical study, 272 GD patients were enrolled to receive different preoperative treatments (ATDs and thyroxine, ATDs and iodine, iodine, no preparation), 74% of whom accepted ATDs and thyroxine (31). Starting with carbimazole (or methimazole), 10 mg *quarter in die (*q.i.d.) or propylthiouracil 100 mg q.i.d. to block the synthesis of thyroid hormone, and then maintain an adequate drug dosage. Then, reducing the doses of ATDs by half and providing patients with thyroxine 0.20 mg qd continued this regimen for at least 4 weeks. The thyroid is close to the normal state after adequate pretreatment, which is beneficial to risk reduction of intraoperative blood loss. Moreover, no thyroid storm postoperatively occurred in the GD patients prepared preoperatively with ATDs, and the hospitalization time was shorter than that in patients who took ATDs and iodine preoperatively (6.5 days versus 18 days), for the latter have more severe hyperthyroidism and need to be hospitalized for observation during preoperative preparation.

For GD patients who decide to choose thyroidectomy, remaining euthyroid and continuously inhibiting TSH secretion are of critical importance. Combining a medium dose of methimazole and L-T4 is also called the “block-and-replace” (BR) method in internal medicine treatment, which can not only maintain euthyroid status but also postpone surgery to a suitable age for pediatric GD patients. In the comparison of practical application before surgery, the incidence of euthyroid was present in 87.4 ± 18.6% of patients with the BR approach, which was better than that of methimazole alone (47.1 ± 30.9%) (32) by one ATD treatment period. Even though the ultimate goals were different from the discipline perspective, the essence of the effect on the thyroid is the same: blocking endogenous thyroid hormone synthesis and inhibiting TSH to maintain the morphology of the thyroid by providing exogenous hormones. This regimen offers GD patients a worth deserving method to achieve a euthyroid state before the surgery safely and quickly, reducing the incidence of complications in the perioperative period and providing a choice for GD patients who are allergic to iodine.

## Preparation under special circumstances

The degree of GD’s thyrotoxicosis is different; sometimes the conventional preoperative preparations mentioned above are unsuitable for patients under special circumstances.

## Hyperthyroid heart disease

Beta-blockers, such as propranolol, are always used to treat GD in higher-risk individuals, including elderly individuals those with severe thyrotoxicosis, and those with existing cardiovascular disease. The combination of iodine solution and beta-blockers was first proposed in 1980, and potassium iodide 60 mg tid was given to patients for 10 days (33) after receiving propranolol, which led to a remarkable decrease in the mean serum levels of thyroxine (T4) and triiodothyronine (T3) to the normal range of thyroid function preoperatively (p<0.001).

## Rapid preoperative preparation

When GD patients choose operation or have surgery in an emergency, they need to be treated slightly complicated. Rapid preoperative treatment could attain effective outcomes similar to common treatment, but time saving (34). Using iodine to treat uncontrolled hyperthyroidism preoperatively is safe and decreases thyroid hormone levels and heart rate. However, doses of Lugol’s solution differ in the literature, making comparisons of optimum dosage difficult (25). Giving glucocorticoids with a stress- dose could solve the low reservation of adrenal glands and prevent conversion from T4 to T3 (35), the premedicated method of antithyroid agents, beta-blockade and corticosteroids have been proven to be effective. A research found that accepting the combination of 500 mg iopanoic acid bid, 1 mg dexamethasone bid, beta-blocker with ATD could help 80% of patients achieve normal levels of T4 and T3 in a week (36).

For patients intolerant to ATDs or patients in urgent need of surgery, the regimen of SSKI 2 drops tid, beta-blockers, dexamethasone 2 mg q.i.d. and cholestyramine 4 g q.i.d. was recommended (37). This concept was recommended continuously by Stefan Fischli et al. in 2016, who concluded that patients could achieve rapid preparation by taking 5% Lugol’s solution 13 drops tid, 2 mg/d dexamethasone and beta-blockers (38). As for medication interval and change in doses, a study summarized in 2020 that giving iodine in 1 hour after thionamide administration is appropriate, while hydrocortisone should be given 100 mg intravenous injection (IV) quaque 8 hora (q8h) the day of surgery, tapering off over 3 days gradually (35).

In the latest guideline of the American Society for Apheresis, therapeutic plasma exchange (TPE) was recommended as a second-line therapy in patients with thyrotoxicosis (39). The advantage of TPE in thyroid dysfunction is that it not only makes thyroid hormone levels fall rapidly, but also decreases antibodies, binding proteins, cytokines and catecholamines. GD patients were treated with ATDs and Lugol’s solution before the TPE procedure, and beta-blocker was added when atrial fibrillation occurred. Statistically significant reductions in FT3 and FT4 and increases in TSH were observed (p =0.003, 0.033 and 0.008,respectively) after TPE (40). Furthermore, TPE could provide a useful back-up to preoperative treatment as a supplement when the combination mentioned above fails to prepare the patient well with giant toxic nodular or methimazole-induced agranulocytosis for thyroidectomy (41). These rapid preoperative preparation regimens are summarized in **Table 2**.

**Table 2:** Rapid preoperative preparation of Graves’ disease in different publications.

Publication	Literature type	Participants	initial regimen/day	ultimate regimen/day	Effect
H. O. Kirkizlar,2022 (41)	Retrospective	Female:10/11 $\bar{\text{y}}$ : 44.91	ATD+LS+ beta-blockers,	TPE+ beta-blockers	FT3↓* FT4↓* TSH↑*
Yusaku Mori, 2021 (36)	Case report	A 21-year-old woman	MMI + Li_2_CO_3_	MMI + Li_2_CO_3_+ DSMS+ iodine, 7 days	euthyroid, surgery underwent
Katarzyna Barwinek,2020 (42)	Case report	A 63-year-old woman	PTU +LS+Li_2_CO_3_ +beta-blockers + DSMS IV	TPE twice	surgery underwent
Adibah Ali, 2019 (34)	Retrospective	Female:18/19 $\bar{\text{y}}$ : 33	LS for 10 days	iopanoic acid +DSMS+ beta-blockers+ thionamides 7 days.	Surgery underwent
Stefan Fischli, 2016 (39)	Prospective	Female:7/10	ATDs	5% LS+DSMS+ beta-blocker, 10-14days	Euthyroid and no complication
Henry B, 2015 (38)	Review	/	/	beta-blockers+ SSKI+ DSMS +cholestyramine	recommended

$\bar{\text{y}}$ , mean or median age; FT3, Free triiodothyronine; FT4, Free thyroxine; TSH, Thyroid Stimulating Hormone; ATD, antithyroid drugs; LS, Lugol’s solution; MMi, Methimazole; Li2CO3, lithium carbonate; TPE, therapeutic plasma exchange; DSMS, dexamethasone; PTU, propylthiouracil; IV, intravenous; ↓, decrease significantly; ↑, increase; *:p<0.01.

## Conclusion

Over many years of exploration in the clinic, the surgical treatment and preoperative preparation of hyperthyroidism have gradually formed some commonly recognized and reasonable programs. However, the specific implementation of the regimens and the effect of preoperative preparation are practically different in the literature reports. Comparisons of different drug combinations preoperatively, the rapid preoperative preparation of Graves’ disease, the treatment method of giant Graves’ disease and other practical clinical problems still need to be further explored.

## Author contributions

Literature search and screening: TX, XZ. Writing: TX, XZ, TW. Surgical and medical practices: TX, XZ, TW. All authors read and approved the final version of the manuscript. All authors contributed to the article and approved the submitted version.

## References

[1] Davies TF Andersen S Latif R Nagayama Y Barbesino G Brito M. Graves' disease. Nat Rev Dis Primers (2020) 6(1):52. doi: 10.1038/s41572-020-0184-y 32616746

[2] Fischer S Ehlert U. Hypothalamic-pituitary-thyroid (HPT) axis functioning in anxiety disorders. A systematic review. Depress Anxiety (2018) 35(1):98–110. doi: 10.1002/da.22692 29064607

[3] Zhou F Wang X Wang L Sun X Tan G Wei W. Genetics, epigenetics, cellular immunology, and gut microbiota: emerging links with graves' Disease. Front Cell Dev Biol (2021) 9:794912. doi: 10.3389/fcell.2021.794912 35059400 PMC8765724

[4] Prunty JJ Heise CD Chaffin DG. Graves' Disease pharmacotherapy in women of reproductive age. Pharmacotherapy (2016) 36:64–83. doi: 10.1002/phar.1676 26799350

[5] Piantanida E. Preoperative management in patients with Graves' disease. Gland Surg (2017) 6:476–81. doi: 10.21037/gs.2017.05.09 PMC5676164 29142837

[6] Liu X Wong CKH Chan WWL Tang EHM Woo YC Lam CLK. Outcomes of graves' Disease patients following antithyroid drugs, radioactive iodine, or thyroidectomy as the first-line treatment. Ann Surg (2021) 273(6):1197–206. doi: 10.1097/SLA.0000000000004828 33914484

[7] Vital D Morand GB Meerwein C Laske RD Steinert HC Schmid C. Early timing of thyroidectomy for hyperthyroidism in graves' Disease improves biochemical recovery. World J Surg (2017) 41(10):2545–50. doi: 10.1007/s00268-017-4052-1 28681142

[8] de Mul N Damstra J Nieveen van Dijkum EJM Fischli S Kalkman CJ Schellekens WM. Risk of perioperative thyroid storm in hyperthyroid patients: a systematic review. Br J Anaesth (2021) 127(6):879–89. doi: 10.1016/j.bja.2021.06.043 34389171

[9] Frank ED Park JS Watson W Chong E Yang S Simental AA. Total thyroidectomy: Safe and curative treatment option for hyperthyroidism. Head Neck (2020) 42(8):2123–8. doi: 10.1002/hed.26148 32199035

[10] Huang S-M Liao W-T Lin C-F Sun HS Chow N-H. Effectiveness and mechanism of preoperative lugol solution for reducing thyroid blood flow in patients with euthyroid graves' Disease. World J Surg (2016) 40:505–9. doi: 10.1007/s00268-015-3298-8 26546192

[11] Ansaldo GL Pretolesi F Varaldo E Meola C Minuto M Borgonovo G. Doppler evaluation of intrathyroid arterial resistances during preoperative treatment with Lugol's iodide solution in patients with diffuse toxic goiter. J Am Coll Surg (2000) 191(6):607–12. doi: 10.1016/S1072-7515(00)00755-9 11129808

[12] Zhu JQ Li ZH Wei T Zhang H Gong RX Xu HZ. [Study on thyroid defunctionalization method for the preoperative preparation of hyperthyroid operation]. Sichuan Da Xue Xue Bao Yi Xue Ban (2007) 38(5):866–70. In Chinese 17953380

[13] Calissendorff J Falhammar H. Lugol's solution and other iodide preparations: perspectives and research directions in Graves' disease. Endocrine (2017) 58:467–73. doi: 10.1007/s12020-017-1461-8 PMC5693970 29075974

[14] Leung AM Braverman LE. Consequences of excess iodine. Nat Rev Endocrinol (2014) 10:136–42. doi: 10.1038/nrendo.2013.251 PMC3976240 24342882

[15] Yilmaz Y Kamer KE Ureyen O Sari E Acar T Karahalli O. The effect of preoperative Lugol's iodine on intraoperative bleeding in patients with hyperthyroidism. Ann Med Surg (Lond) (2016) 9:53–7. doi: 10.1016/j.amsu.2016.06.002 PMC4932873 27408715

[16] Whalen G Sullivan M Maranda L Quinlan R Larkin A. Randomized trial of a short course of preoperative potassium iodide in patients undergoing thyroidectomy for Graves' disease. Am J Surg (2017) 213:805–9. doi: 10.1016/j.amjsurg.2016.07.015 27769543

[17] Randle RW Bates MF Long KL Pitt SC Schneider DF Sippel RS. Impact of potassium iodide on thyroidectomy for Graves' disease: Implications for safety and operative difficulty. Surgery (2018) 163(1):68–72. doi: 10.1016/j.surg.2017.03.030 29108701 PMC5736456

[18] Lindner K Kußmann J Fendrich V. Preoperative potassium iodide treatment in patients undergoing thyroidectomy for graves' Disease-perspective of a european high-volume center. World J Surg (2020) 44:3405–9. doi: 10.1007/s00268-020-05593-0 32447416

[19] Tsai CH Yang PS Lee JJ Liu TP Kuo CY Cheng SP. Effects of preoperative iodine administration on thyroidectomy for hyperthyroidism: A systematic review and meta-analysis. Otolaryngol Head Neck Surg (2019) 160(6):993–1002. doi: 10.1177/0194599819829052 30721111

[20] Mercier F Bonal M Fanget F Maillard L Laplace N Peix JL. Does surgery without lugol's solution pretreatment for graves' Disease increase surgical morbidity? World J Surg (2018) 42(7):2123–6. doi: 10.1007/s00268-017-4443-3 29302725

[21] Al Jassim A Wallace T Bouhabel S Majdan A Hier M Forest VI. A retrospective cohort study: do patients with graves' disease need to be euthyroid prior to surgery? J Otolaryngol Head Neck Surg (2018) 47(1):37. doi: 10.1186/s40463-018-0281-z 29784035 PMC5963139

[22] Barranquero AG Muñoz de Nova JL Gómez-Ramírez J Valdés de Anca Á Porrero B Blanco Terés L. Effect of preoperative potassium iodide administration on Graves' disease surgery: a propensity score analysis. Am J Surg (2021) 222(5):959–63. doi: 10.1016/j.amjsurg.2021.04.023 33941360

[23] Kamijo K. Clinical studies on potassium iodide-induced painless thyroiditis in 11 graves' Disease patients. Intern Med (2021) 60:1675–80. doi: 10.2169/internalmedicine.6411-20 PMC8222119 33431733

[24] Mooij CF Cheetham TD Verburg FA Eckstein A Pearce SH Léger J. 2022 European Thyroid Association Guideline for the management of pediatric Graves' disease. Eur Thyroid J (2022) 11(1):e210073. doi: 10.1530/ETJ-21-0073 34981748 PMC9142815

[25] Calissendorff J Falhammar H. Rescue pre-operative treatment with Lugol's solution in uncontrolled Graves' disease. Endocr Connect (2017) 6:200–5. doi: 10.1530/EC-17-0025 PMC5434745 28325735

[26] Ross DS Burch HB Cooper DS Greenlee MC Laurberg P Maia AL. 2016 american thyroid association guidelines for diagnosis and management of hyperthyroidism and other causes of thyrotoxicosis. Thyroid (2016) 26(10):1343–421. doi: 10.1089/thy.2016.0229 27521067

[27] Erbil Y Ozluk Y Giriş M Salmaslioglu A Issever H Barbaros U. Effect of lugol solution on thyroid gland blood flow and microvessel density in the patients with Graves' disease. J Clin Endocrinol Metab (2007) 92(6):2182–9. doi: 10.1210/jc.2007-0229 17389702

[28] Sharma A Stan MN. Thyrotoxicosis: diagnosis and management. Mayo Clin Proc (2019) 94:1048–64. doi: 10.1016/j.mayocp.2018.10.011 30922695

[29] Cheetham T Bliss R. Treatment options in the young patient with Graves' disease. Clin Endocrinol (Oxf) (2016) 85:161–4. doi: 10.1111/cen.12871 26252256

[30] Doubleday AR Sippel RS. Hyperthyroidism. Gland Surg (2020) 9:124–35. doi: 10.21037/gs.2019.11.01 PMC7082267 32206604

[31] Heimann P Martinson J. Surgical treatment of thyrotoxicosis: results of 272 operations with special reference to preoperative treatment with anti-thyroid drugs and L-thyroxine. Br J Surg (1975) 62:683–8. doi: 10.1002/bjs.1800620903 51660

[32] Vigone MC Peroni E Di Frenna M Mora S Barera G Weber G. "Block-and-replace" treatment in Graves' disease: experience in a cohort of pediatric patients. J Endocrinol Invest (2020) 43(5):595–600. doi: 10.1007/s40618-019-01144-0 31713721

[33] Feek CM Sawers JS Irvine WJ Beckett GJ Ratcliffe WA Toft AD. Combination of potassium iodide and propranolol in preparation of patients with Graves' disease for thyroid surgery. N Engl J Med (1980) 302(16):883–5. doi: 10.1056/NEJM198004173021602 6892650

[34] Ali A Debono M Balasubramanian SP. Outcomes after urgent thyroidectomy following rapid control of thyrotoxicosis in graves' Disease are similar to those after elective surgery in well-controlled disease. World J Surg (2019) 43:3051–8. doi: 10.1007/s00268-019-05125-5 31407090

[35] Himes CP Ganesh R Wight EC Simha V Liebow M. Perioperative evaluation and management of endocrine disorders. Mayo Clin Proc (2020) 95:2760–74. doi: 10.1016/j.mayocp.2020.05.004 33168157

[36] Mori Y Hiromura M Terasaki M Kushima H Ohara M Fukui T. Very rare case of Graves' disease with resistance to methimazole: a case report and literature review. J Int Med Res (2021) 49(3):300060521996192. doi: 10.1177/0300060521996192 33682498 PMC7944538

[37] Panzer C Beazley R Braverman L. Rapid preoperative preparation for severe hyperthyroid Graves' disease. J Clin Endocrinol Metab (2004) 89:2142–4. doi: 10.1210/jc.2003-031981 15126532

[38] Burch HB Cooper DS. Management of graves disease: A review. JAMA (2015) 314:2544–54. doi: 10.1001/jama.2015.16535 26670972

[39] Fischli S Lucchini B Müller W Slahor L Henzen C. Rapid preoperative blockage of thyroid hormone production / secretion in patients with Graves' disease. Swiss Med Wkly (2016) 146:w14243. doi: 10.4414/smw.2016.14243 26765838

[40] Connelly-Smith L Dunbar NM. The 2019 guidelines from the American Society for Apheresis: what's new? Curr Opin Hematol (2019) 26:461–5. doi: 10.1097/MOH.0000000000000534 31577607

[41] Kirkizlar HO Celik M. Therapeutic plasma exchange in hyperthyroidism prior to surgery. J Endocrinol Invest (2023) 46(1):173–9. doi: 10.1007/s40618-022-01897-1 35963982

[42] Barwinek K Gąsior-Perczak D Trepka S Szczodry A Kopczyński J Sitarz-Żelazna Z. Effective preoperative plasmapheresis treatment of severe hyperthyroidism in a patient with giant toxic nodular goiter and methimazole-induced agranulocytosis. Medicina (Kaunas) (2020) 56(6):290. doi: 10.3390/medicina56060290 32545570 PMC7353859

